# On the Origin and Development of the Sacs and Pulps of the Human Teeth

**Published:** 1841-12

**Authors:** 


### *On the Origin and Development of the Sacs and Pulps of the Human Teeth.* By John Goodsir, Jr. Surgeon, Anstruther, Fifeshire.

In the January number for 1839, of the "Edinburgh Medical and Surgical Journal," is an able and well written paper on the above named subject by Mr. Goodsir, who has succeeded in discovering the rudiments of the teeth at a much earlier period than has been done by any former odontologist. He is of the opinion that they originate from mucous membrane, and that those of the secondary set, have no connection with the first. "Dentition," he says, "commences by the formation of the primitive dental groove, on the floor of which the rudiments of the pulps of the milk-teeth appear as globular or conical papillæ; septa afterwards pass from the outer to the inner side of the groove, between the papillæ, and thus each of the latter becomes situated in an open-mouthed follicle, which is the primitive condition of the future sac." He also tells us, that the germ of the first superior temporary molaris is the first of the rudiments of the milk-teeth that appears, and that this is discoverable some time between the sixth and seventh week of embryonic life. "It is at this period," says he, "a simple, free granular papilla, like many others on the surface of the mucous membrane and skin." Anterior to this, and in the same jaw, at about the eighth week, he has succeeded in discovering a second papilla, which is the germ of the cuspidatus or canine tooth.

The ridge within which is the groove that contains these papilla, advances in an "indistinct manner to the median line, during the ninth week," and on each side of this line, "an oblong papillæ with a notched lamina in front of it, and immediately afterwards another smaller papillæ, external to the former; these last papillæ," says he, "are the germs of the incisive teeth, and are placed in connection with the inter-maxillary system." The progress of the incisive papillæ during the tenth week is slow, "their anterior laminæ only increasing somewhat in size." From the sides of the groove within which the germs of the primitive teeth are situated, processes shoot across and come together before and behind the rudiment of the first temporary molaris, enclosing it in a follicle, through the opening or mouth of which the papilla is observable. A like follicle is slowly formed around the germ of the cuspidatus. At about this period, the germ of the second primitive molaris, shows itself in the form of a small papilla, at the side, and seemingly "a production of the rounded lobule, which terminates posteriorly to the outer ridge." The papilla of the incisores advance constantly during the eleventh and twelfth weeks, and septæ pass between them during this time, so that they become sunk as it were, each in a separate and well defined follicle. The germ of the anterior molaris undergoes but little apparent change, while that of the posterior enlarges, and by the gradual folding of the terminal lobule, which the author describes, becomes situated in a follicle like the other papillæ. Behind this, the primitive groove extends itself a short distance, within which the first permanent molaris first shows itself.

During the thirteenth week, the follicle of the second milk molar becomes more fully developed, and the rudimentary papillæ of the other teeth begin to assume the forms of the crowns of the teeth which they are respectively to form, the papillæ grow faster than the follicles, and protrude slightly from their mouths; the follicles however increase, so as soon to enclose the germs of the teeth, and thus become their matrices or sacs. From the time the matrices close, the papillæ of the first teeth become gradually moulded into the shape of the teeth that are formed from them. As the formative process goes on, "each branch of the dental artery, as it arrives at the fundus of its destined sac, sends off a number of radiating twigs, which run in the substance of the cellular submucous tissue, (which constitutes the outer membrane of the sac) towards the gum, from which others proceed to inosculate with them. By "the combined twigs" of these, the true membrane of the sac is minutely ramified, while not the smallest fillet is sent to the granular substance. After sending off these, the dental branch "divides into a number of contorted ramifications between the base of the pulp and the sac, which from smaller ramuscule are transmitted to the pulp itself." In the meantime, the germs of the first secondary molares are formed together with their follicles, within which they have become completely inclosed, and granular matter deposited within their sacs; the edges of the walls of the groove of these teeth adhere, but the walls themselves do not, by which a cavity of some size is left below the tooth, "or between it and the gum." "This cavity," says Mr. GOODSIR, "is a reserve of delicate mucous membrane," and from which the materials for the second and third permanent molares, are furnished.

He denies, as we have before remarked, that the permanent teeth have their origin from the temporary ones, and contends that they, like these, have their origin from mucous membrane, having no connection with the others whatever. His researches have been conducted upon an extensive scale, and certainly throw much new and valuable light upon this department of odontology.—*Balt. Ed.*

☞The foregoing notice was furnished to the editors of the Maryland Medical and Surgical Journal, from which publication it is copied.—*Balt. Ed.*

